# Central sterile supply departament management on hospital-associated infections: a systematic review and meta-analysis

**DOI:** 10.1590/S1678-9946202567016

**Published:** 2025-03-03

**Authors:** Jing Shuai, Maoyu Liu, Jialing Hou, Yu Chen, Jun Jiang, Jing Yu, Liang Yin

**Affiliations:** 1 The General Hospital of the PLA Western Theater Command, Central Sterile Supply Department, Chengdu, Sichuan, China; 2 The General Hospital of the PLA Western Theater Command, Pain Management Department, Chengdu, Sichuan, China

**Keywords:** Hospital-associated infections, Sterile supply center management, Systematic review, Meta-analysis, Infection control, Adverse events

## Abstract

Hospital-associated infections (HAIs) pose significant risks in clinical settings, and sterile supply centers management plays a crucial role in infection control. This systematic review and meta-analysis aimed to evaluate the impact of supply center management on the incidence of HAIs and adverse events. The systematic review encompassed studies that compared supply center management protocols with standard care. The PRISMA guidelines were followed to search seven databases for relevant studies. The meta-analysis calculated pooled odds ratios (OR) for HAIs and adverse events, and heterogeneity was assessed using Tau^2^, Chi-squared, and I^2^ statistics. Eight studies were included in the final analysis, each assessing intervention efficacy. The results revealed a significant reduction in HAIs (pooled OR=0.3; 95%CI [0.19; 0.49]). Adverse events were also significantly reduced (pooled OR=0.15; 95%CI [0.09; 0.25]). Heterogeneity was low for both HAIs (Tau^2^=0.00; I^2^=0%) and adverse events (Tau^2^=0.04; I^2^=19%), which indicated a consistent effect across the studies. Sterile supply center management significantly reduced the incidence of HAIs and adverse events. This suggests these interventions are effective in improving clinical outcomes and could be a vital component of infection control strategies in healthcare facilities.

## INTRODUCTION

Sterile supply center management is crucial for infection control within healthcare settings^1^. Proper handling, storage, and distribution of medical supplies, instruments, and equipment are essential for preventing hospital-associated infections (HAIs) and maintaining a safe environment for patients, healthcare workers, and visitors^2^.

Inadequate sterilization processes in healthcare facilities can lead to several adverse events, including contamination from incorrectly sterilized equipment, malfunction of medical devices post-sterilization, and healthcare-associated infections (HAIs) resulting from non-sterile instruments. Such adverse events show significant risks to patients’ health, contributing to negative clinical outcomes and undermining infection control efforts within healthcare facilities^3-5^.

According to WHO, approximately seven out of every 100 patients in high-income countries and 15 out of every 100 patients in low- and middle-income countries acquire hospital-associated infections (HAIs) during their hospital stay^6^ and studies have reported an HAI prevalence ranging from 8% to 58% in India^7,8^. HAIs are associated with considerable economic burdens due to extended hospital stays, increased morbidity, and additional healthcare costs^9^. Proper Central sterile supply department (CSSD) management requires meticulous cleaning, disinfection, and sterilization processes. The primary aim is to eliminate pathogens and maintain equipment sterility, which directly impacts infection control in healthcare settings. Nosocomial infections, also known as HAIs, are a significant challenge to healthcare delivery in the world. These infections not only prolong hospital stays but also increase emotional distress, lead to long-term disability, and, in severe cases, result in patient mortality. The economic burden of HAIs is substantial, affecting both patients and healthcare providers.

The centralization of these processes via Central Sterile Supply Departments (CSSD) has emerged as a crucial strategy in many hospitals^10^. The concept of CSSD emerged in 1928, following a recommendation by the American College of Surgeons to centralize surgical supplies and dressings. Nowadays, CSSD are a dedicated hub for processing medical and surgical instruments^8^. CSSD are self-contained units, equipped to receive, clean, pack, disinfect, sterilize, store, and distribute sterile instruments according to strict protocols and standards. The primary goal is to ensure the availability of sterile items within the healthcare facility, optimizing cost-efficiency without compromising patient care or healthcare provider productivity^2^. Strategic placement of the CSSD is crucial. Ideally located close to nursing units, labor and delivery suites, and operating theatres, it facilitates an efficient workflow. A minimum space of seven square feet per hospital bed is recommended for CSSD allocation, enabling future expansion^11^.

The CSSD encompasses distinct functional areas: collection, decontamination, cleaning, assembling, packing, sterilization, storing and distribution of sterile goods, and administrative spaces^4^. The core responsibilities include processing used and contaminated supplies, ensuring a constant supply of sterile items, contributing to infection control, and offering expertise and instruction to hospital staff regarding the sterilization of supplies and equipment^12^.

Effective sterile supply center management hinges on rigorous protocols for cleaning, sterilizing, and storing medical supplies and instruments. Following standardized sterilization methods, such as steam sterilization, ethylene oxide sterilization, and low-temperature sterilization, is paramount. Proper packing and labelling are vital to maintaining sterility until use^13^.

The physical CSSD layout impacts efficiency and hygiene. Clear segregation of clean and contaminated areas, as well as proper storage practices, are crucial to prevent cross-contamination. Maintaining appropriate inventory levels and implementing rotation systems ensures timely use and inspection of supplies^14^.

Quality assurance is paramount in sterile supply center management^15^. Regular audits, environmental monitoring, and thorough documentation of sterilization processes and supply usage are essential for identifying potential areas of improvement and adhering to best practices and regulatory standards^4,15^. Comprehensive training programs are crucial for upholding CSSD standards. Equipping personnel with the necessary knowledge and skills is vital for maintaining a safe and effective sterile supply department^16^.

Effective sterile supply centers management play a crucial role in preventing HAIs within healthcare facilities^3,14-16^. Maintaining a sterile environment and implementing effective supply center management has gained significant attention in recent years due to its direct impact on patient safety and healthcare outcomes. This systematic review and meta-analysis aimed to evaluate the existing literature on CSSD management and its correlation with HAIs’ incidence.

## MATERIALS AND METHODS

### Eligibility criteria

This systematic review strictly adhered to the Preferred Reporting Items for Systematic Reviews and Meta-Analyses (PRISMA) guidelines^17^. The PECO framework guided this study’:

- **Population**: Patients who were admitted to healthcare facilities.
- **Exposure**: Implementation of supply center management protocols.
- **Comparator**: Healthcare facilities without supply center management protocols.
- **Outcome**: Primary outcome measures included the incidence of healthcare-associated infections (HAIs) and adverse events. Adverse events include: unqualified cleaning quality; device assembly errors; identification defects; sterilization mode errors; supply delay; occupational exposure; accidents; standardized management; nosocomial infection during instrument use; damage and loss of instruments and items, and incidence of sharp instrument injury^18^ ^,^ ^19^.

In these studies, standard care refers to common and conventional infection control and sterilization methods that have been implemented without using advanced management models such as Plan-Do-Check-Act (PDCA). The inclusion criteria were 1) peer-reviewed randomized controlled trials and observational studies; 2) all patient demographics that were admitted to healthcare facilities; 3) studies examining the effect of supply center management protocols; 4) report of incidence of HAIs and adverse events, and 5) studies published in English with no timeframe limitation. Exclusion criteria were: 1) reviews, editorials, commentaries, and non-peer-reviewed literature; 2) studies focusing on outpatients or non-hospital settings; 3) studies not addressing supply center management protocols; 4) studies that were not reporting specific outcomes related to HAIs or adverse events; and 5) studies not available in English.

### Database search strategy

A comprehensive search strategy was employed across multiple electronic databases to identify relevant studies. The search strategy employed Boolean operators and Medical Subject Headings (MeSH) terms to enhance search precision and breadth. PubMed, Embase, Web of Science, Cochrane Central Register of Controlled Trials (CENTRAL), Scopus, CINAHL, and PsycINFO databases were searched. Chart 1 shows the search strategy, combining MeSH terms and keywords.

**Chart 1:** Search strategies used across the assessed databases.

Database	Search string
PubMed	“Healthcare-Associated Infections”[MeSH Terms] AND “Central Service”[MeSH Terms] AND (“Sterilization”[MeSH Terms] OR “Disinfection”[MeSH Terms]) AND “supply management” AND “hospital” NOT “editorial” NOT “commentary”
Embase	(‘hospital infection’/exp OR ‘nosocomial infection’:ab,ti) AND (‘disinfection’/exp OR ‘sterilization’/exp) AND (‘central supply, hospital’/exp OR ‘supply centre management’:ab,ti) NOT [letters]/lim NOT [notes]/lim
Web of Science	TS=((“Healthcare-Associated Infections” OR “Hospital-Acquired Infections” OR “Nosocomial Infections”) AND (“Supply Centre Management” OR “CSSD”) AND (“Sterilization” OR “Disinfection”)) NOT TS=(“commentary” OR “editorial”)
Cochrane Central	(“Hospital Infections”:ti,ab,kw OR “Nosocomial Infections”:ti,ab,kw) AND (“Sterilization”:ti,ab,kw OR “Disinfection”:ti,ab,kw) AND (“Supply Centre Management”:ti,ab,kw OR “Central Sterile Supply Department”:ti,ab,kw)
Scopus	(TITLE-ABS-KEY (“hospital acquired infection” OR “healthcare associated infection” OR “nosocomial infection”) AND TITLE-ABS-KEY (“sterilization” OR “disinfection”) AND TITLE-ABS-KEY (“central sterile supply department” OR “CSSD” OR “supply centre management”)) NOT (EXCLUDE (PUBYEAR, 2023))
CINAHL	(MH “Cross Infection+”) AND (MH “Sterilization+”) OR (MH “Disinfection+”) AND (TX “central supply services department” OR TX “supply centre management”) NOT (MH “Commentary” OR MH “Editorial”)
PsycINFO	(DE “Hospital-Acquired Infections” OR DE “Nosocomial Infections”) AND (DE “Sterilization” OR DE “Disinfection”) AND (DE “Hospital Supply Management” OR DE “Central Sterile Supply Department”) NOT (DE “Commentary” OR DE “Editorial”)

### Data extraction process

A standardized data extraction form was developed and rigorously pilot-tested on a subset of selected studies to ensure comprehensive data capture. The initial screening process involved reviewing the titles and abstracts of all identified studies to determine their relevance to the research question. Studies that met the inclusion criteria were assessed in full text for final eligibility. Two independent reviewers extracted data from each study to mitigate bias and ensure reliability. Disagreements were resolved via consensus or consultation with a third reviewer. Extracted data included author(s), year of publication, study location, study design, sample size, patient demographics, intervention/control details, outcome measures, and results. Methodological quality indicators such as randomization, blinding, and attrition rates were also recorded. In instances in which numerical data were not explicitly stated, the reviewers contacted the studies corresponding authors to request the necessary information. If data remained unavailable, the study was excluded, and the impact of this exclusion was assessed during analysis.

### Assessment of bias

The ROBINS-E tool^20^ (Risk of bias in non-randomized Studies) of exposures (ns-e tool) was used to assess the risk of bias in the studies.

### Statistical analysis protocol

A retrospective meta-analysis was conducted using the Review Manager (RevMan) software (version 5.4.1, The Cochrane Collaboration, London, United Kingdom). The primary outcomes were the odds of HAIs and incidence of adverse events associated with CSSD management protocols. The included studies were retrospective case-control studies providing sufficient data for calculating odds ratios (ORs) with 95% confidence intervals (CIs). A random-effects (RE) model was employed due to anticipated clinical and methodological heterogeneity among studies. Funnel plots were created to assess potential publication bias for each outcome.

### Quality assessment and certainty of evidence

In addition to the ROBINS-E tool for bias assessment, the Grading of Recommendations Assessment, Development, and Evaluation (GRADE) approach^21^ was employed to assess evidence certainty across the studies. Two independent reviewers applied the GRADE criteria to evaluate evidence quality for each outcome, considering factors such as risk of bias, inconsistency, indirectness, imprecision, and publication bias.

## RESULTS

### Study selection process

**Figure 1:**
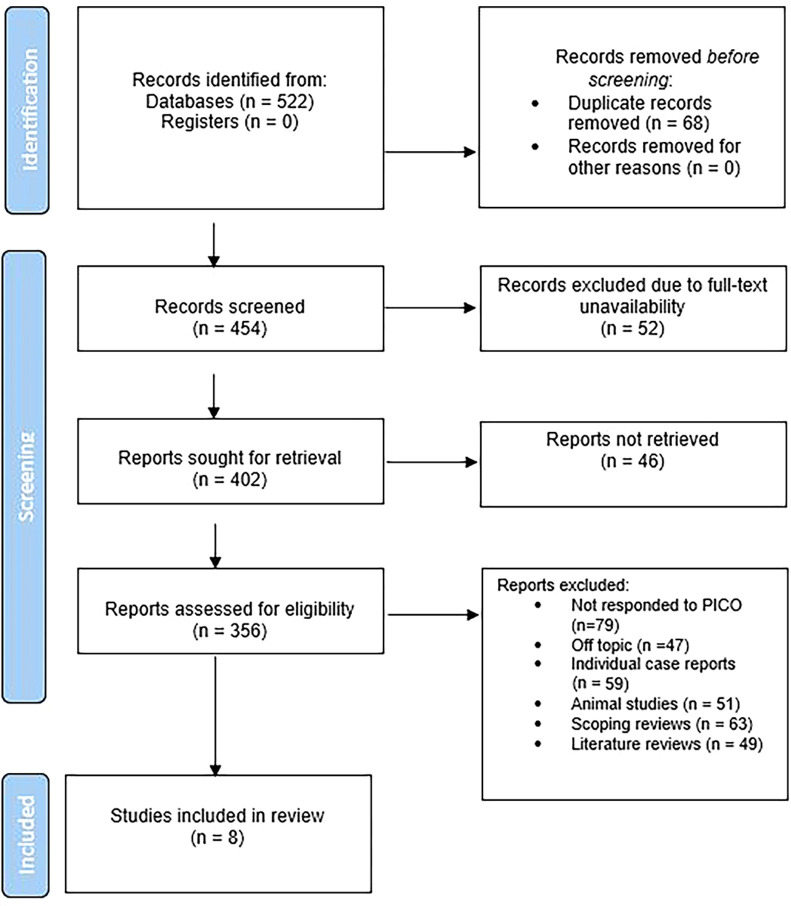
Flow chart of the screening process.

The study selection process (Figure 1) began with an initial database search that yielded 522 records. After eliminating 68 duplicates, 454 records remained for screening. Due to access issues, 52 records were excluded, leaving 402 records for further consideration. Of these, 46 records were not obtained for detailed review. The remaining 356 records underwent an in-depth review. During this phase, 47 records were irrelevant to the research question, while 79 records did not meet the predefined PECO criteria. Additionally, 49 narrative reviews, 51 animal studies, and 59 scoping reviews were excluded, as they did not align with our inclusion criteria. After this rigorous screening, eight studies^5,18,19,22-25^ were identified as suitable for inclusion in the systematic review.

### Bias assessment

**Figure 2:**
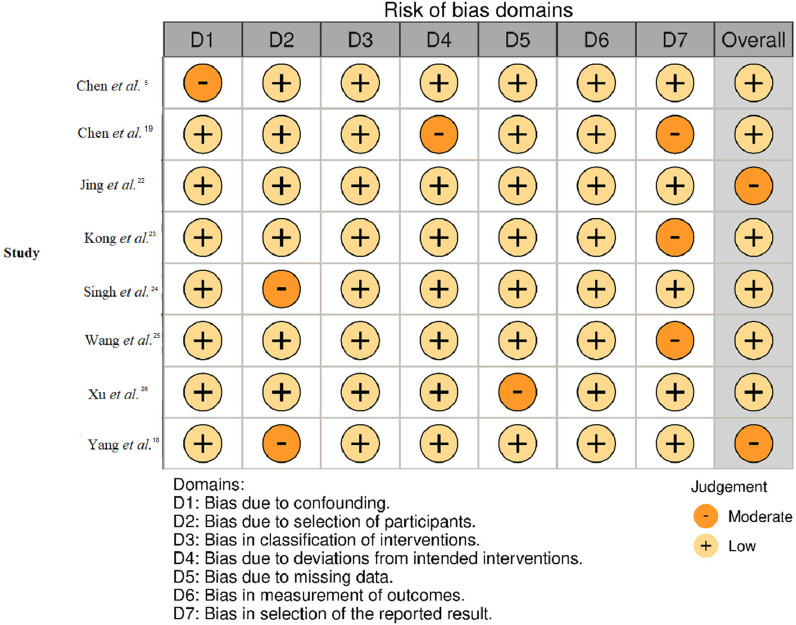
Bias assessment across different domains.

The bias assessment revealed that most studies had a low risk of bias in most domains (Figure 2). For confounding (D1), two studies^5,24^ showed a moderate risk, whereas the remaining studies showed a low risk. Selection of participants (D2) was another domain that had a moderate risk in two studies^5,18^ and low risk in the rest. The classification of interventions (D3) uniformly showed a low risk of bias across all studies. Deviations from intended interventions (D4) were predominantly low risk, but showed occasional moderate risk in two studies^19,24^. Missing data (D5) were low in all but one study^26^, which had a moderate risk. Outcomes measurement (D6) maintained a low risk, except for two instances^19,25^ that showed a moderate risk. Finally, selection of reported result (D7) had moderate risk in three studies^19,23,25^ and low risk in the others.

### Demographic characteristics

Chart 2 summarizes the key characteristics of the included studies^5,18,19,22-25^. Chen *et al*.^19^ conducted a comparative analysis to evaluate conventional vs. research-based management approaches in a sterile supply center, involving 300 participants over two phases. Jing *et al*.^22^ performed a controlled comparison of 562 medical devices under different management protocols over a one-year period, highlighting differences in outcomes. Kong *et al.* ^23^ assessed the impact of the PDCA cycle on 1,020 patients, comparing a control group with a PDCA-implemented group over two years. Singh *et al*.^24^ conducted an observational study with 11 CSSD staff members, focusing on internal practices and outcomes within a single group. Wang *et al*.^25^ and Xu *et al*.^26^ explored the effects of specific management protocols on health personnel and patients, respectively, with a focus on comparing control and intervention groups. Finally, Yang *et al.* ^18^ investigated the impact of management protocols on medical staff outcomes over a one-year period.

**Chart 2:** Characteristics of the included studies.

Article	Study duration	Group description	Sample size (n)	Interventions	Main findings	Statistical significance (P-value)
Chen *et al.* ^5^	Nov 2020 Feb 2021 (Conventional) Mar 2021 Jun 2021 (Research)	Conventional Group vs. Research Group	300 (150 per group)	Conventional infection management vs. PDCA cycle management with risk factor management	Lower detection rate of Gram-negative bacillus and Gram-positive cocci in the research group Lower incision infection rate in the research group Lower total incidence of irregular events in the research group Higher disinfection rate of object surface, hands of medical staff, and air in the research group Improved nursing quality scores in the research group	<0.05
Chen *et al.* ^19^	Jan 2020 Dec 2022	Retrospective Study	101 adverse events	Convenience sampling approach for adverse events in CSSD	Most adverse events: substandard cleaning quality, faulty assembly, defective marking Inspection and packing, and device cleaning were the highest risk chains for adverse events Factors influencing adverse events: type of person who is responsible, education, years of work experience, device structure, number of instruments in the kit, kit size	<0.05
Jing *et al*.^22^	Jan 2020 Jun 2020 (Control) Jul 2020 Dec 2020 (Observation)	Control Group vs. Observation Group	562 medical devices (282 control, 280 observation)	Conventional process vs. Management quality sensitive index guided by key point control theory	Higher process index scores (cleaning, assembly, labelling, and sterilization) in the observation group Lower incidence of wet packing Shorter service times (replenishment, retrieval, and preparation) in the observation group	<0.05
Kong *et al.* ^23^	Jan 1^st^, 2019 Dec 31^st^, 2019 (Control) Jan 1^st^, 2020 Dec 31^st^, 2020 (PDCA)	Control Group vs. PDCA Group	1,020 patients (508 control, 512 PDCA)	Conventional management vs. PDCA cycle quality management	Lower incidence of nosocomial infections in the PDCA group Higher pass rates for medical staff knowledge of nosocomial infection and hand hygiene Higher rate of endoscope cavity disinfection Lower incidence of sharp injury and biological pollution in PDCA group	Nosocomial infections: 0.002 <br> Other indexes: <0.05
Singh *et al.* ^24^	Feb 2018 Apr 2018	Single Group Observational	CSSD staff: 11	Workflow management in CSSD	Effective sterilization (steam and gas) with use of indicators Contribution to the reduction of nosocomial infections Maintenance of quality standards for sterilization and disinfection	
Wang *et al.* ^25^	Sep 2015 Apr 2016 (Control) Jul 2016 Feb 2017 (Observation)	Control Group vs. Observation Group	176 health personnel (86 control, 90 observation)	Traditional management vs. Sub-specialties management model	Higher satisfaction scores in the observation group Lower complaint rates about device errors Lower damage rates to specialist medical devices Improved theoretical knowledge and practical skills of CSSD professionals	
Yang *et al.* ^18^	Jan 2020 Dec 2020	Medical staff in hospital CSSD (Control vs. Observation)	32 (16 control, 16 observation)	Standardized management vs. Defect management improvement based on JCI standard	Higher cleaning quality rate, infection awareness, standard implementation, hand hygiene, and scores for theoretical knowledge and practical ability in observation group Lower incidence of adverse events Higher satisfaction scores in observation group Satisfaction related to training, education, and title	P<0.05
Xu *et al.* ^26^	Jan 2022 - Apr 2022 (Common) May 2022 - Dec 2022 (PDCA)	Patients who are receiving digestive endoscopy care	Common: 90 PDCA: 156	Application of PDCA cycle on nursing quality management and risk control	Decreased infection rate from 4.44% to 0.64% Increased qualified air rate from 92.22% to 98.72% Improvement in physiological parameters and nursing satisfaction Higher qualified rates for endoscopic cavity and external disinfection Increased management scores post-PDCA	Not specified

PDCA = Plan-Do-Check-Act; CSSD = Central sterile supply department; JCI = Joint Commission International.

### Assessed interventions and observed statistical significance

In the studies, standard care refers to common and conventional infection control and sterilization methods that have been implemented without using advanced management models such as PDCA. Chen *et al*.^5^ compared methods of traditional infection management with a PDCA cycle approach that included risk factor management, with statistical results showing significant improvements in the PDCA group. Chen *et al*.^19^ used non-random sampling to identify adverse events in the CSSD and observed a significant reduction. Jing *et al*.^22^ evaluated the impact of a Management Quality Sensitive Index based on key point control theory compared to traditional management processes, showing notable improvements in management processes. Kong *et al*.^23^ demonstrated the PDCA management method led to a substantial reduction in hospital-associated infections compared to conventional management. Wang *et al*.^25^ and Xu *et al*.^26^ investigated the effects of specialized management models and the PDCA cycle on improving nursing quality and risk control, respectively, both finding significant results. In contrast, Singh *et al*.^24^ and Yang *et al*.^18^ focused on workflow management and standardization. However, sufficient data for statistical interpretation were not provided.

### Overall assessments

Chen *et al*.^5^ reported that the research group experienced a lower detection rate of Gram-negative bacilli and Gram-positive cocci compared to the control group, indicating an effective reduction in potential pathogens. Additionally, the research group showed a lower incision infection rate, suggesting the intervention was beneficial in reducing surgical site infections. The study also found a lower total incidence of irregular events in the research group, implying improvement in overall clinical safety. Furthermore, the research group achieved higher qualified rates of disinfection of object surfaces, hands of medical staff, and air quality, which are critical factors in infection control and prevention. Chen *et al*.^19^ identified most adverse events as stemming from substandard cleaning quality, faulty instrument assembly, and defective labelling. This highlights the importance of meticulous attention to detail in the sterilization and preparation processes within CSSDs. Their findings also suggest inspection and packing, along with device cleaning, were the highest risk chains for adverse events, indicating critical control points that require stringent management. Factors influencing adverse events included the type of person that was responsible, their level of education, years of experience, device structure, the number of instruments in the kit, and the kit size, underscoring the multifaceted nature of risk factors in clinical settings.

Jing *et al*.^22^ observed higher process index scores (cleaning, assembly, labelling, sterilization) in the observation group, indicating that the intervention led to improvements in these key areas. The study also reported a lower incidence of wet packing, a common issue that can compromise instruments sterility, and shorter service times for replenishment, retrieval, and preparation, indicating enhanced efficiency in supply center management. Kong *et al.* ^23^ found a lower incidence of nosocomial infections in the PDCA group compared to the control group, demonstrating the effectiveness of the PDCA cycle in infection control. Higher pass rates for medical staff’s knowledge of nosocomial infection and hand hygiene were reported, signifying an improvement in staff education and practices. The PDCA group also reported a higher rate of endoscope cavity disinfection and a lower incidence of sharp injury and biological pollution, indicating safer clinical practices and environments.

Singh *et al.* ^24^ concluded that effective sterilization, both steam and gas, contributed to the reduction of nosocomial infections and maintained quality standards for sterilization and disinfection. This emphasizes the critical role of sterilization processes in preventing infections. Wang *et al*.^25^ reported higher satisfaction scores, lower complaint rates about device errors, and lower damage rates to medical devices in the observation group, suggesting the intervention improved the reliability and safety of medical equipment. The study also noted improved theoretical knowledge and practical skills among CSSD professionals, which likely contributed to the observed enhancements in service quality.

Xu *et al*.^26^ reported a decrease in the infection rate from 4.44% to 0.64% and an increase in the qualified air rate from 92.22% to 98.72%, indicating significant improvements in clinical outcomes and environmental quality. Improvements in physiological parameters, nursing satisfaction, and qualified rates for endoscopic cavity and external disinfection were also noted. The increase in management scores post-PDCA further supports the effectiveness of this cyclical quality improvement process. Yang *et al.* ^18^ found a higher quality cleaning rate, infection awareness, standard implementation, hand hygiene, and higher scores for theoretical knowledge and practical ability in the observation group. The lower incidence of adverse events and higher satisfaction scores further indicate that the intervention had a positive impact on the healthcare setting. Satisfaction was linked to factors such as training, education, and professional title, suggesting comprehensive education and professional development are essential components of successful infection control strategies.

### Meta-analysis of efficacy

**Figure 3:**
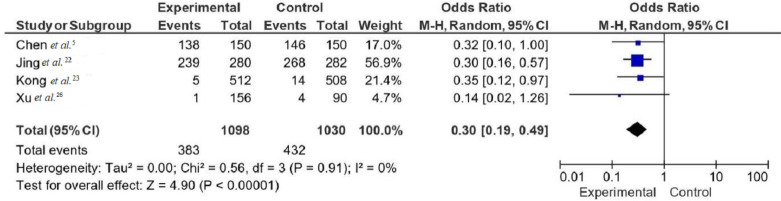
Forest plots of the efficacy of sterile supply center management protocols in reducing the incidence of HAIs.

Figure 3 shows a forest plot that depicts the OR from four studies to evaluate the efficacy of supply center management in reducing the incidence of HAIs^5,22,23,26^. Only these four articles were included in the meta-analysis as data were not heterogeneous and therefore pooled OR was robust. The pooled OR across these studies was 0.30 (95%CI: 0.19–0.49), demonstrating a statistically significant reduction in the odds of HAIs for groups using supply center management protocols compared to controls. There was no significant heterogeneity among the studies (I^2^=0%, Tau^2^=0.00, Chi-squared=0.56, FD=3, P=0.91), suggesting consistent effect sizes across different clinical settings. The overall effect was highly significant (Z=4.90, P<0.00001), confirming the effect of supply center management protocols on reducing the incidence of HAIs.

### Meta-analysis of adverse events

**Figure 4:**
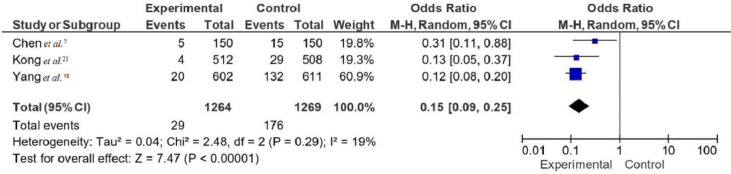
Forest plots of adverse events associated with sterile supply center management protocols.

Figure 4 shows a forest plot that depicts the OR from three studies to assess the efficacy of supply center management in reducing adverse events^5,18,23^. The types of adverse events include unqualified cleaning quality, device assembly errors, identification defects, sterilization mode errors, supply delay, occupational exposure, accidents, standardized management, nosocomial infection during instrument use, damage and loss of instruments and items, and the incidence of sharp instrument injury^18,19^. The pooled OR across these studies was 0.15 (95%CI: 0.09-0.25), indicating a statistically significant reduction in the odds of adverse events for groups using supply center management protocols. There was a low level of heterogeneity among the studies (I^2^=19%, Tau^2^=0.04, Chi-squared=2.48, FD=2, P=0.29). The overall effect was highly significant (Z=7.47, P<0.00001), confirming the effect of supply center management protocols on reducing adverse events.

### Publication bias

The funnel plot for the efficacy of supply center management showed no evidence of publication bias, with studies evenly distributed around the vertical line. This suggests the published studies are a fair representation of the overall evidence. The study by Chen *et al*.^5^ showed high precision and no association between the exposure and the outcome, whereas Kong *et al*.^23^ and Yang *et al*.^18^ showed low precision and an association between the exposure and the outcome.

### GRADE assessment observations

The selected trials^5,18,19,22-25^ initially assigned a low certainty due to their observational design. Across the eight studies, common findings included improvements in infection control practices and healthcare process outcomes, such as lower infection rates, higher disinfection pass rates, and increased satisfaction scores among healthcare staff and patients. The risk of bias for these studies was assessed as low to moderate, reflecting some concerns about the validity of the findings. However, details on other GRADE considerations such as inconsistency, indirectness, imprecision, and other potential biases were not reported, limiting a comprehensive assessment of these dimensions.

## DISCUSSION

Studies have shown implementing Plan-Do-Check-Act (PDCA) cycle management can lead to significant reductions in infection rates and enhance the overall quality of healthcare. Specifically, it has been reported that this approach helps decrease nosocomial infections while also improving disinfection and sterilization rates^5,23^.

Similar risk factors and processes within the CSSD that contribute to adverse events have been identified by both Chen *et al*.^19^ and Jing *et al*.^22^, however, their analyses differ in scope. Chen *et al*.^19^ focused on specific types of adverse events, such as substandard cleaning quality and faulty assembly, whereas Jing *et al*.^22^ provided a more process-oriented analysis, highlighting improvements in cleaning, assembly, labelling, and sterilization processes. The comparative study by Chen *et al*.^19^ supports previous findings that research-based management approaches can reduce adverse events. Similarly, the implementation of the PDCA cycle in studies by Kong *et al*.^23^ and Xu *et al*.^26^ aligns with the literature suggesting continuous improvement cycles enhance infection control and overall management efficiency in healthcare settings.

The importance of effective sterilization processes and quality standards in reducing nosocomial infections and improving service quality has been emphasized in various studies^24,25^. For instance, Singh *et al*.^24^ highlighted these factors, whereas Wang *et al*.^25^ expanded their analysis by examining the impact of interventions on healthcare professionals’ satisfaction and complaint rates related to medical devices.

The successful implementation of PDCA cycles has been reported to lead to improvements in infection control, higher rates of qualified disinfection, and increased staff satisfaction. These findings suggest the PDCA cycle is an effective tool for continuous quality improvement in diverse healthcare settings, as demonstrated by Xu *et al*.^26^ and Yang *et al*.^18^. Comparative studies highlight the effectiveness of the PDCA cycle in infection management, as observed in Chen *et al*.^5^ and Kong *et al*.^23^, aligning with the literature that supports continuous quality improvement in infection control. Furthermore, the Management Quality Sensitive Index introduced by Jing *et al.* ^22^ corroborates the previous findings on structured quality management frameworks enhancing CSSD efficiency.

The focus of Singh *et al*.^24^ on the effectiveness of sterilization methods, specifically steam and gas, in reducing nosocomial infections, distinguishes their study from others. While other studies addressed broader aspects of infection control and service quality, Singh *et al*.^24^ provided a more detailed analysis of sterilization effectiveness, offering insights into the specific impact of these sterilization techniques.

The protocols’ ability to reduce HAIs implies that their widespread adoption could improve patient safety and decrease associated healthcare costs. The high significance of the overall effect supports their integration into standard practice. Supply center management protocols can be a critical component of quality improvement initiatives within healthcare institutions. Continuous monitoring and evaluation are necessary to ensure sustained effectiveness and identify areas for improvement.

Prior research has investigated various approaches to enhance safety management within the CSSD. Meticulous record-keeping of sterilization processes helps reinforce safety controls by enabling consistent monitoring of daily practices^27^. Instrument tracking systems adopted in CSSDs in China and elsewhere have reduced packing errors for surgical instruments, preventing omissions and mismatches^28,29^.

The PDCA cycle, a management approach developed by Dai Ming, involves four iterative steps: plan, do, check, and act. This cyclical process helps improving management effectiveness and completeness by continuously evaluating and refining plans^30-32^. In the context of endoscopic procedures, the PDCA cycle has been effective in refining nursing care plans and enhancing nursing care quality^33,34^.

Adhesion to established rules and regulations ensures staff compliance with codes of conduct and diminishes the occurrences of high-risk events. Due to the high humidity, elevated temperatures, and limited airflow in CSSD environment, which can facilitate bacterial growth and impact equipment cleanliness and staff comfort, it is vital to monitor environmental parameters such as temperature, humidity, lighting, and ventilation. Ensuring that these indicators meet standards helps to maintain an appropriate microclimate and provides staff with protective measures to lessen environmental burdens^35^.

### Limitations

While comprehensive in its synthesis of existing data, the study has limitations. The reliance on published literature may have introduced publication bias, as studies with positive outcomes are more likely to be published than those with negative or inconclusive results, potentially overestimating the efficacy of supply center management protocols. Furthermore, the study’s observational design precluded the ability to establish causality. While significant associations were found between supply center management protocols and reduced incidence of HAIs and adverse events, unmeasured confounding factors could have influenced the results.

### Practice-based recommendations

Our findings support the following recommendations made for healthcare organizations:

1. Implement supply center management protocols: The significant decrease in HAIs and adverse events demonstrates the effectiveness of these protocols, which should be incorporated into standard operations.
2. Use the PDCA cycle: The consistent benefits of the PDCA cycle for healthcare service improvement make it a valuable tool for quality improvement programs.
3. Address specific risk factors and processes: Healthcare organizations should use targeted interventions to mitigate these issues.

The studies highlight the importance of high sterilization and disinfection standards in lowering nosocomial infections and enhancing service quality. Healthcare providers should ensure strict compliance with sterilization protocols and consider the broader effects on staff satisfaction and care quality.

The successful application of PDCA cycles in continuous quality improvement processes emphasizes their value in strengthening infection control measures, improving disinfection rates, and boosting staff satisfaction. Considering the findings on sterilization methods, healthcare establishments should assess the effectiveness of their sterilization techniques and consider adopting or refining steam and gas sterilization methods to further diminish nosocomial infection rates.

## CONCLUSION

The study concluded that sterile supply center management protocols significantly reduced the incidence of HAIs and adverse events in clinical settings. The aggregated data from various research efforts provided strong evidence for the efficacy of these protocols, as evidenced by the substantial decrease in odds ratios for both HAIs and adverse events in experimental groups compared to control groups. The consistency of the effect sizes across diverse clinical environments suggests these protocols could be generalized to different healthcare contexts with similar beneficial outcomes. The low heterogeneity among the studies further supports this conclusion, indicating that the findings were consistent despite potential variations in study design, population, and healthcare settings. The study also highlighted the effectiveness of the PDCA cycle in continuous improvement of sterile supply center management, as it was properly applied and tailored to the specific needs of the healthcare facility.
